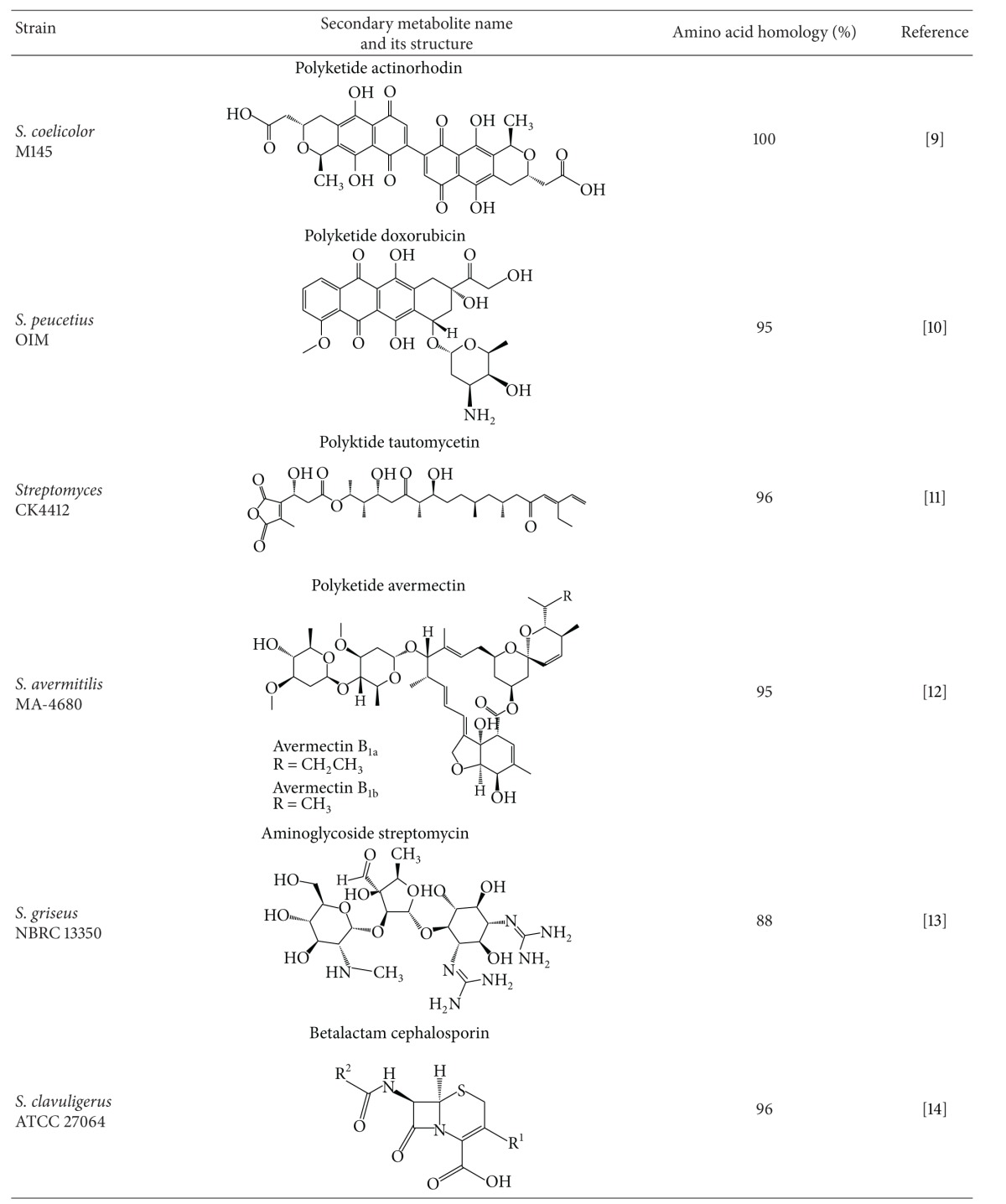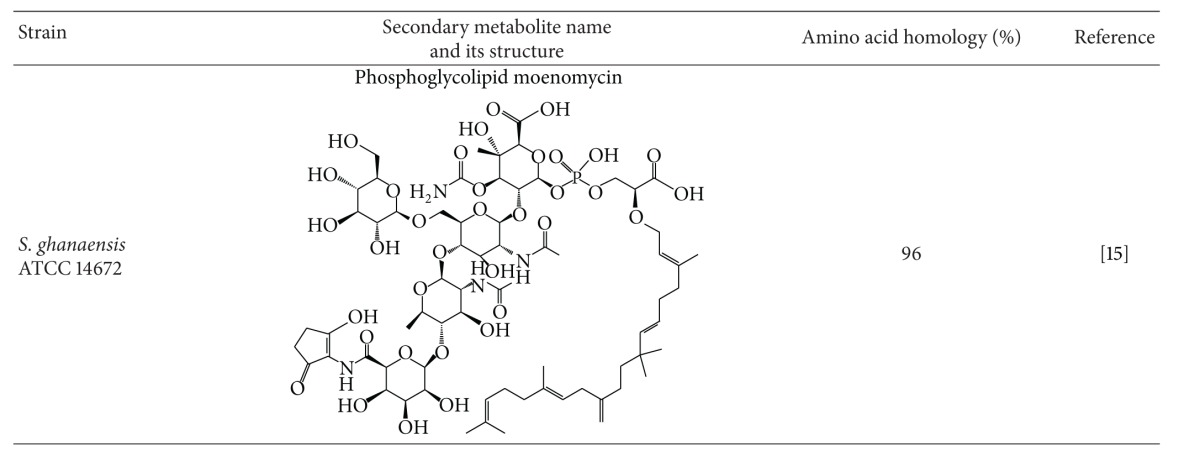# Identification and Biotechnological Application of Novel Regulatory Genes Involved in *Streptomyces* Polyketide Overproduction through Reverse Engineering Strategy

**DOI:** 10.1155/2013/549737

**Published:** 2013-03-04

**Authors:** Ji-Hye Nah, Hye-Jin Kim, Han-Na Lee, Mi-Jin Lee, Si-Sun Choi, Eung-Soo Kim

**Affiliations:** Department of Biological Engineering, Inha University, Incheon 402-751, Republic of Korea

## Abstract

Polyketide belongs to a family of abundant natural products typically produced by the filamentous soil bacteria *Streptomyces*. Similar to the biosynthesis of most secondary metabolites produced in the *Streptomyces* species, polyketide compounds are synthesized through tight regulatory networks in the cell, and thus extremely low levels of polyketides are typically observed in wild-type strains. Although many *Streptomyces* polyketides and their derivatives have potential to be used as clinically important pharmaceutical drugs, traditional strain improvement strategies such as random recursive mutagenesis have long been practiced with little understanding of the molecular basis underlying enhanced polyketide production. Recently, identifying, understanding, and applying a novel polyketide regulatory system identified from various Omics approaches, has become an important tool for rational *Streptomyces* strain improvement. In this paper, DNA microarray-driven reverse engineering efforts for improving titers of polyketides are briefly summarized, primarily focusing on our recent results of identification and application of novel global regulatory genes such as *wblA*, SCO1712, and SCO5426 in *Streptomyces* species. Sequential targeted gene manipulation involved in polyketide biosynthetic reguation synergistically provided an efficient and rational strategy for *Streptomyces* strain improvement. Moreover, the engineered regulation-optimized *Streptomyces* mutant strain was further used as a surrogate host for heterologous expression of polyketide pathway.

## 1. Introduction: *Streptomyces* Polyketide Biosynthesis and Pathway-Specific Regulation

 The high G+C Gram-positive filamentous soil bacteria* Streptomyces* are well known for their superior characteristics in producing a variety of secondary metabolites, including many pharmaceutically valuable compounds such as antibiotics, anticancer agents, and immunosuppressants [1, 2]. These secondary metabolites are commonly synthesized by biosynthetic enzymes, whose corresponding genes are typically clustered in the *Streptomyces* chromosome and are proposed to be under tight, complicated regulation at the transcriptional level [3]. Among *Streptomyces* secondary metabolites are polyketides, which belong to one of the largest natural product families [4]. *Streptomyces *polyketide biosynthesis is initiated by the key enzyme(s) referred to as polyketide synthase (PKS), which is typically classified by 3 different types. Type I PKSs are multifunctional enzymes whose domains are arranged into several modules, each of which controls incorporation of a specific precursor unit into a growing polyketide backbone during chain elongation, generating macrolide compounds such as erythromycin, tylosin, avermectin, amphotericin, and tautomycetin [5]. Type II PKSs are multienzyme complexes that perform a single set of enzymatic activities for iterative biosynthesis of aromatic products including actinorhodin, tetracycline, and doxorubicin [6]. Type III PKSs are homodimeric enzymes that catalyze iterative condensation reactions, typically known as chalcone synthase-like PKSs [7]. The genes, enzymes, and the mechanism of *Streptomyces* polyketide biosynthesis have been thoroughly reviewed previously [8]. In general, a simple carboxylic acid starter unit such as acetyl-CoA or propionyl-CoA is transferred to the cysteine active site of a *β*-ketoacyl synthase (KS) in the PKS complex. A specific extender unit determined by an acyl transferase (AT) domain in the PKS complex, such as a malonyl-CoA, methylmalonyl-CoA, or ethylmalonyl-CoA, is transferred to the thiol group of the phosphopantetheine arm of an adjacent acyl carrier protein (ACP). These subunits are joined by a decarboxylative condensation catalyzed by KS and remain covalently attached to ACP, followed by no, partial, or full series of keto group processing reactions involving *β*-ketoacyl reductase (KR), dehydratase (DH), and enoyl reductase (ER). The growing chain is transferred to a downstream module of type I or iteratively recycled in type II and III for further rounds of elongation and processing to produce a full-length polyketide chain. The completed chain is typically released from the PKS by a terminal thioesterase (TE) to form a macrocyclic lactone ring or an aromatic compound. 

Although a general molecular-level mechanism for polyketide biosynthesis has been relatively well characterized, a comprehensive understanding of the complex polyketide regulatory networks in *Streptomyces* species is yet to be elucidated [3]. The biosynthesis of *Streptomyces* polyketide is regulated *via* multiple regulatory pathways induced by both nutritional and environmental stimuli [17, 18]. While various global regulatory systems present in most *Streptomyces* species are known to control both morphological differentiation and polyketide production, polyketide biosynthetic gene sets are subject to pathway-specific regulation by linked regulatory genes [19–21]. Some pathway-associated regulatory genes encode specialized types of regulatory protein, such as “SARPs” (*Streptomyces* antibiotic regulatory proteins) often associated with genes for type II aromatic polyketide biosynthesis [22, 23] and “LALs” (large ATP-binding regulators of the LuxR family) associated with some gene sets for type I macrolide polyketide biosynthesis [16]. Most of these pathway-specific regulatory genes are transcriptionally regulated by various global regulatory networks in most *Streptomyces* species, but their detailed mechanisms remain largely unknown.

## 2. Reverse Engineering Approaches Applied in *Streptomyces* Strain Improvement

 Since all genome sequences of the first model species, *Streptomyces coelicolor*, became publically available around the year 2002, various Omics-guided strategies have been applied to increase the understanding of *Streptomyces* global regulatory networks involved in polyketide biosynthesis [24, 25]. Although most pathway-specific regulatory genes have been identified on the basis of their typical location within the biosynthetic pathway gene cluster, global regulatory genes are much more difficult to identify even in the well-characterized *S. coelicolor* because of the presence of more than 300 annotated putative regulatory open reading frames (ORFs) in the genome sequences [24, 25]. Recently, Omics-guided reverse engineering approaches have emerged as an effective tool for investigating gene expression alterations associated with polyketide overproduction in several *Streptomyces* industrial strains [26]. This strategy enabled us to compare the recursively and randomly mutagenized overproducing industrial mutant (OIM) with the wild-type (WT) strain at the molecular gene level, eventually leading us to identify previously unknown primary and/or secondary metabolic genes critical for secondary metabolite overproduction. The *S. coelicolor* DNA microarray system was successfully applied to the erythromycin-overproducing *Saccharopolyspora erythraea* OIM as well as tylosin-overproducing *S. fradiae* OIM strains [26]. A similar genome-wide transcriptome analysis also showed that a pleiotropic antibiotic regulator, *afsS* in *S. coelicolor *A3(2), and various AfsS-dependent genes are regulated by various nutritional stress responses [27]. *S. peucetius *DNA microarray analysis also revealed that expression patterns of *S. peucetius *genes involved in doxorubicin production change under different culture conditions [28]. In addition, genomics-driven approaches were applied to stimulate cryptic pathways such as 51-membered glycosylated macrolides, stambomycins [29, 30]. Chemical perturbation of secondary metabolism also demonstrated important links to primary metabolism, implying that small molecules could also enhance yields of secondary metabolites for discovery and biochemical characterization [31]. 

## 3. Identification and Manipulation of a Novel Regulatory Gene *wblA* in *S. coelicolor*

 A putative negative regulatory gene involved in polyketide biosynthesis, SCO3579, was originally proposed as a *whiB*-like putative transcription factor gene referred to as *wblA* in* S. coelicolor* [33]. *whiB* is one of five key regulatory genes including *whiA, whiB, whiG, whiH, *and *whiI* and known to be required for early stages of the transition of aerial hyphae to spores. Moreover, the *whiB* mutant exhibits poorly septated aerial hyphae with long and tightly coiled phenotype, and its expression is proposed to be repressed by a transcription factor BldD. [33]. Although *whiB* is a developmental regulatory gene identified and characterized in *S. coelicolor* as being essential for the sporulation of aerial hyphae, the biological function of *wblA *with regard to secondary metabolite regulation has not yet been examined. During the search for previously unknown polyketide regulatory genes, *wblA* was identified as a novel antibiotic downregulatory gene in a *Streptomyces* reverse-engineering approach [9]. The recursively mutated doxorubicin-overproducing *S. peucetius* OIM and the wild type *S. peucetius *subsp*. caesius *ATCC 27952 were examined for potential transcriptional differences between the 2 strains. After systematic analyses of growth phase-dependent transcription profiles, 20 genes with particularly large transcriptional changes between the 2 strains were selected and individually overexpressed in *S. coelicolor* under the control of the strong promoter of the *Streptomyces* expression vector. Among these genes, overexpression of *wblA* inhibited actinorhodin biosynthesis in *S. coelicolor*, and the transcript encoded by an actinorhodin-specific activator gene was reduced in *wblA*-overexpressing *S. coelicolor* [9]. These results suggest that *wblA* is a broadly functioning downregulatory gene for polyketide biosynthesis in *Streptomyces* species. It was suggested that WblA, which contains 4 conserved cysteine residues, may be sensitive to redox changes, perhaps *via* disulfide bond formation as has been found for the *E. coli *OxyR transcription factor [32, 34]. Recently, transcriptome analysis with *S. coelicolor *microarray approach in a *wblA *mutant exhibited that approximately 180 genes involved in primary metabolism and actinorhodin biosynthesis and 100 genes related to the aerial hyphal growth were overexpressed and underexpressed, respectively [9]. WblA was then proposed to be important in the slow-down of biomass accumulation, the change from aerial hyphal initial cells to the subapical stem, apical compartments that precede sporulation, and oxidative stress response similar to that of *Corynebacterium glutamicum* WhcA [35]. WhcA in *C. glutamicum* was proved to physically bind to a specific protein named SpiA (stress protein interacting with WhcA) only under the nonoxidative condition. Interestingly, a SpiA ortholog present in *S. coelicolor* also showed a similar WblA-SpiA interaction in the oxidative stress response in *S. coelicolor*, and a global regulatory protein called AdpA (A-factor-dependent protein A) might control the *wblA* expression through direct binding to the promoter region of *wblA* (Lee et al., unpublished data). Currently, several *wblA* ortholog genes have been identified in various *Streptomyces* species, and they are presumably involved in secondary metabolite regulation (Table 1).

**Figure 1:**
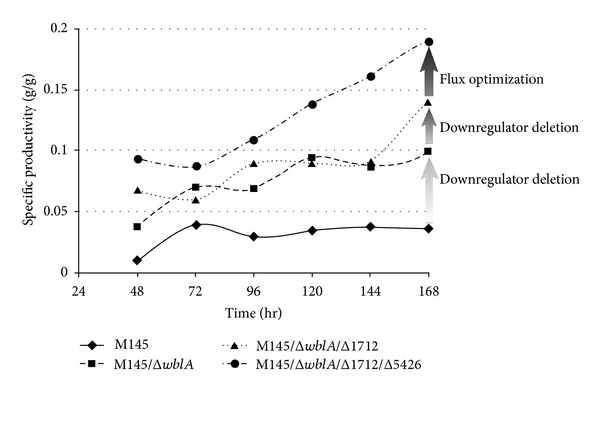
Time-dependent ACT volumetric productivities of wild-type *S. coelicolor* M145 (♦), *S. coelicolor *Δ*wblA* (■), *S. coelicolor* Δ*wblA*ΔSCO1712 (▲), and *S. coelicolor* Δ*wblA*ΔSCO1712Δ5426 (●) cultured in modified R5 media during 9 days in a 2-liter bioreactor [16].

In addition to identifying *wblA*, comparative microarray analysis revealed that SCO1712 expression was considerably lower in actinorhodin overproducing *S. coelicolor *M145 than in the actinorhodin less-producing *S. coelicolor* J1501 [36]. SCO1712 encodes a 205-amino acid protein with an N-terminal TetR-family helix-turn-helix (HTH) DNA-binding domain, whose biological function related to secondary metabolite regulation has not been reported. A significant decrease in the blue pigment actinorhodin was observed in the SCO1712-expressing *S. coelicolor* in plate culture. Moreover, the transcription level of actinorhodin pathway-specific *actII-ORF4* was significantly increased from the *S. coelicolor* M145ΔSCO1712, while an opposite transcription pattern was observed in the SCO1712-overexpressing *S. coelicolor* M145 strain, implying that SCO1712 had a global inhibitory effect on polyketide biosynthesis in *S. coelicolor* [36]. Interestingly, the expression of not only *wblA* but also SCO1712 complemented the Δ*wblA* mutant phenotype independently. These results suggest that *wblA* may not be required for SCO1712 to downregulate polyketide biosynthesis. SCO1712 was additionally disrupted in an *S. coelicolor* M145Δ*wblA* mutant strain; the *S. coelicolor* M145Δ*wblA*ΔSCO1712 double knock-out mutant strain; exhibited considerably higher actinorhodin volumetric productivity (Figure 1). This implies that SCO1712 is not directly related to *wblA *function and more likely encodes a *wblA*-independent polyketide downregulator [36]. These results suggest that sequential targeted gene disruptions of independently working downregulatory genes may be an efficient and rational strategy for *Streptomyces *strain improvement. 

## 4. Identification and Engineering of *wblA* Ortholog in *S. peucetius *Industrial Mutant

To examine whether the *wblA* ortholog is also present in other *Streptomyces* strains with similar biological significance in polyketide regulation, a total genomic DNA library from the doxorubicin-producing *S. peucetius* OIM was constructed. Cosmid library construction and screening successfully generated one positive candidate containing the entire *wblA* ortholog gene (*wblA *_*spe*_) [10]. Sequence analysis of 345 bp ORF containing *wblA *_*spe*_ showed a protein-coding sequence showing a high degree of amino acid similarity with the translated products of *wblA* genes from several previously reported *Streptomyces* genomes, including *S. coelicolor* (95%), *S. avermitilis* MA-4680 (92%), *S. griseus *NBRC 13350 (96%), and *S. clavuligerus* ATCC 27064 (91%). As expected, approximately 35% more doxorubicin and 150% more 14-deoxydoxorubicin (daunorubicin) were produced by *S. peucetius *OIMΔ*wblA *_*spe*_ compared with the *S. peucetius *OIM [10].

**Figure 2:**
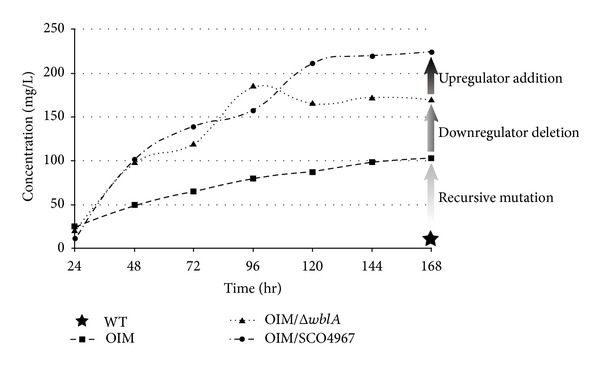
Time-dependent DXR/DNR/aklavinone volumetric productivities of *S. peucetius* WT (★), *S. peucetius* OIM (■), *S. peucetius *OIMΔ*wblA *_*spe*_ (▲), and *S. peucetius *OIMΔ*wblA *_*spe*_/SCO4967 (●) [32].

Through a second round of microarray analysis between *S. peucetius* OIM and OIMΔ*wblA *_*spe*_, six genes showing more than 4-fold transcriptional changes between these two strains were identified, followed by expression in *S. coelicolor *[10]. Since the only *S. coelicolor* exconjugant containing the SCO4967 construct produced approximately 2-fold more actinorhodin than the control, the *Streptomyces* expression vector pSET152 derivative containing SCO4967 was reintroduced into* S. peucetius* OIMΔ*wblA *_*spe*_. SCO4967 is listed to encode a conserved hypothetical protein with little information of *in vivo* biological function. SCO4967 overexpression in *S. peucetius* OIMΔ*wblA *_*spe*_ resulted in approximately 1.7-fold more aklavinone (another doxorubicin precursor polyketide) productivity than that of *S. peucetius* OIMΔ*wblA *_*spe*_ exconjugant harboring an empty vector [10]. Moreover, the SCO4967-containing *S. peucetius* OIMΔ*wblA *_*spe*_ strain exhibited the highest total volumetric production of doxorubicin/daunorubicin/aklavinone, which was approximately 1.9-fold and 1.3-fold higher than those of *S. peucetius *OIM and *S. peucetius* OIMΔ*wblA *_*spe*_, respectively (Figure 2). This implies that sequential genetic manipulation of target genes identified through interspecies comparative microarray analysis may be an efficient and rational strategy for *Streptomyces* strain improvement [10, 37]. 

## 5. Identification and Engineering of *wblA* Ortholog in *Streptomyces *sp. CK4412

 To isolate another *wblA* ortholog gene from a type I polyketide-producing *Streptomyces* species, a total genomic DNA library from *Streptomyces* sp. CK4412 was screened using PCR degenerate primers based on the highly conserved regions present in both the *wblA *sequences of *S. coelicolor* A3(2) and *S. avermitilis *ATCC31780 [11, 38]. *Streptomyces* sp. CK4412 has been known to produce an unusual linear polyketide compound named tautomycetin (TMC), which inhibits T cell proliferation at concentrations 100-fold lower than those needed to achieve maximal inhibition with cyclosporin A. Since TMC is believed to specifically block tyrosine phosphorylation of intracellular signal mediators downstream of Src tyrosine kinases in a T cell-specific manner, TMC is a novel potent T cell-specific immunosuppressive agent whose mechanism of action is different from that of cyclosporin A or FK506. [39–41]. Through *Streptomyces* sp. CK4412 DNA cosmid library screening, one positive cosmid containing the entire *wblA* ortholog gene (named *wblA *_*tmc*_) was selected. Complete sequence analysis revealed that the 390-bp *wblA *_*tmc*_ gene encodes a 130-amino acid protein with a high degree of amino acid similarity with that of the translated *wblA* gene products from *S. coelicolor* (96%), *S. avermitilis* MA-4680 (93%), *S. griseus *NBRC 13350 (89%), and *S. clavuligerus* ATCC 27064 (93%), all of which contain 4 conserved cysteine residues and a helix-turn-helix (HTH) motif [11].

**Figure 3:**
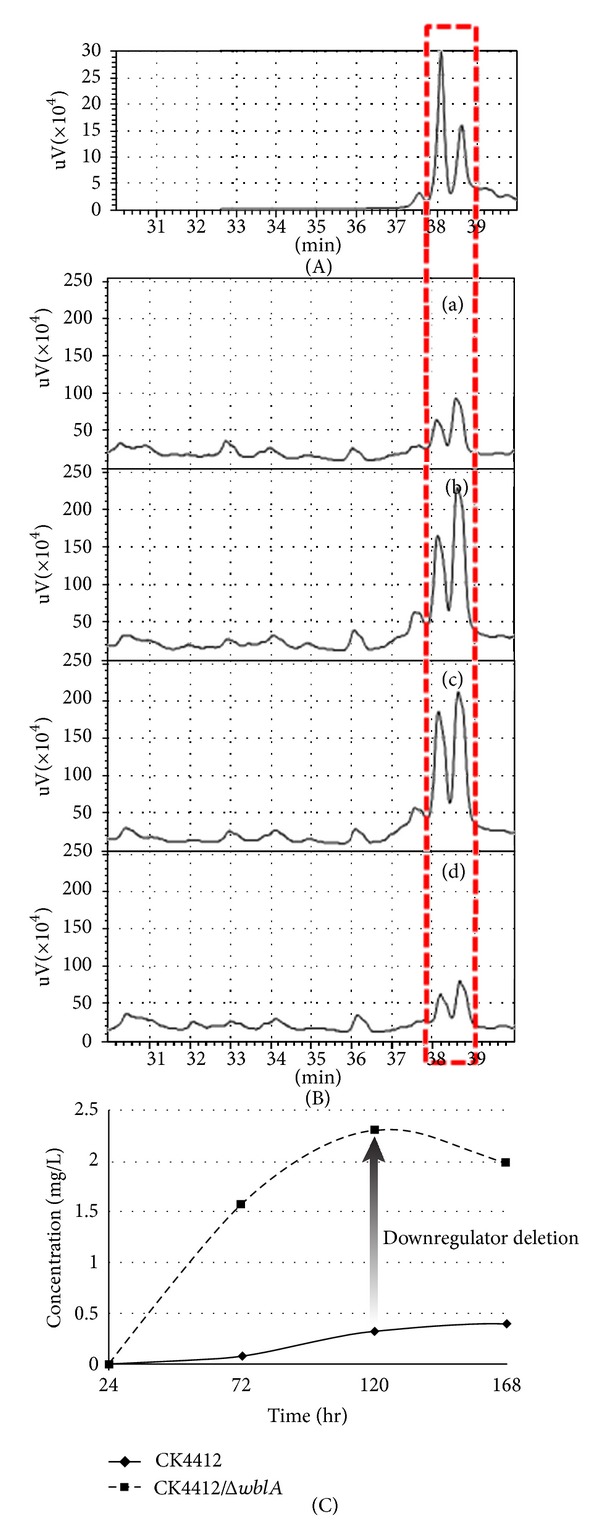
(A) Authentic TMC standard. (B) TMC volumetric productivities measured by quantitative HPLC analyses of the ethyl acetate-extracted culture broths were 1.69 mg · L^−1^ for the wild-type strain CK4412 (a), 5.44 mg · L^−1^ for the *wblA-tmc* disruptant (b), 4.04 mg · L^−1^ for CK4412-001/*wblA-tmc *(c), and 1.41 mg · L^−1^ for CK4412/*wblA-tmc *(d). (C) Time-dependent tautomycetin volumetric productivities of *Streptomyces. *sp. CK4412WT (♦) *S. *sp. CK4412*/*Δ*wblA *_*tmc*_ (■) [36].

 Although *in silico* sequence analyses of *wblA *_*tmc*_ from *Streptomyces* sp. CK4412 was consistent with its putative regulatory roles in other *Streptomyces* polyketide biosyntheses, the *in vivo* function of *wblA *_*tmc*_ was confirmed using a gene disruption approach. Construction of the *wblA *mutant (*Streptomyces* sp. CK4412-001) was generated using PCR-targeted disruption followed by PCR analysis confirmation. Culture broths of *Streptomyces* spp. CK4412, CK4412-001, CK4412-001/*wblA *_*tmc*_, and CK4412/*wblA *_*tmc*_ grown in MS media were extracted, these were analyzed using an antifungal bioassay, and the presence of TMC was quantified using high-pressure liquid chromatography (HPLC). *Streptomyces *CK4412-001 produced approximately 3-fold more TMC compared to WT *Streptomyces *CK4412 (Figure 3). Significantly enhanced antifungal activity against *Aspergillus niger* was also observed in the extracts of *Streptomyces* sp. CK4412-001 under the same culture conditions. Moreover, an integrating conjugative vector, into which the coding region of *wblA *_*tmc*_ and its own upstream promoter region were cloned, was constructed (pSETHYG*wblA*). Both HPLC and a bioassay confirmed that TMC productivity and antifungal activity were reduced in the *Streptomyces* sp. CK4412-001 mutant strain carrying pSETHYG*wblA *back to the WT level, implying that *wblA *_*tmc*_ also plays a global antibiotic downregulatory role in type I polyketide biosynthesis in *Streptomyces* sp. CK4412 (Figure 3). 

## 6. Synergistic Redesign of Polyketide and Precursor Flux Regulatory Pathways 

Comparative transcriptome analysis between *S. coelicolor* WT and an *S. coelicolor* Δ*wblA*ΔSCO1712 double knock-out mutant revealed an additional 14 genes that displayed no particular (less than 1.2-fold) transcriptional changes [42]. These putative *wblA*/SCO1712-independent genes include a carbon flux-regulating SCO5426, which is one of the 3 6-phosphofructokinase genes. SCO5426 disruption was previously reported to enhance both precursor carbon flux and NADPH supply for polyketide biosynthesis by activating the pentose phosphate pathway, resulting in significantly enhanced actinorhodin production in *S. coelicolor *[43]. Based on the above observations, additional deletion of SCO5426 in the *S. coelicolor* Δ*wblA*ΔSCO1712 double knock-out mutant may further enhance actinorhodin precursor flux as well as NADPH supply in *S. coelicolor*. While all mutant strains exhibited comparable growth patterns, the *S. coelicolor* Δ*wblA*ΔSCO1712ΔSCO5426 triple knock-out mutant strain exhibited the highest actinorhodin productivity, which was 1.7-fold and 1.3-fold higher than those of the single knock-out *S. coelicolor* Δ*wblA* and the double knock-out *S. coelicolor* Δ*wblA*ΔSCO1712 mutant strains, respectively [42]. These results suggest that sequential targeted gene disruption of independently working downregulators as well as precursor flux downregulators involved in polyketide biosynthesis may synergistically provide an efficient and rational strategy for *Streptomyces *strain improvement.

## 7. Potential Application as a Surrogate Host for Synthetic Biology

As a complementary strategy for valuable *Streptomyces* polyketide production, functional expression of the target polyketide pathway in a *Streptomyces* heterologous host has been applied. Several secondary metabolite pathways were expressed in relatively well-characterized and genetically amenable *Streptomyces* surrogate hosts including *S. albus, S. lividans, S. coelicolor, S. avermitilis, S. ambofaciens, S. roseosporus*, and *S. grisefuscus* [44, 45]. Recently, the genomes of these strains have been further engineered to maximize foreign polyketide production by deleting some endogenous biosynthetic gene clusters and/or preventing diversion of precursors into competing secondary metabolic pathways. Especially, a genome-minimized *S. avermitilis* industrial mutant strain was also used successfully for foreign polyketide pathway expression as a heterologous expression host [46], suggesting a realistic alternative strategy for overproducing exogenous natural and unnatural polyketides.

**Figure 4:**
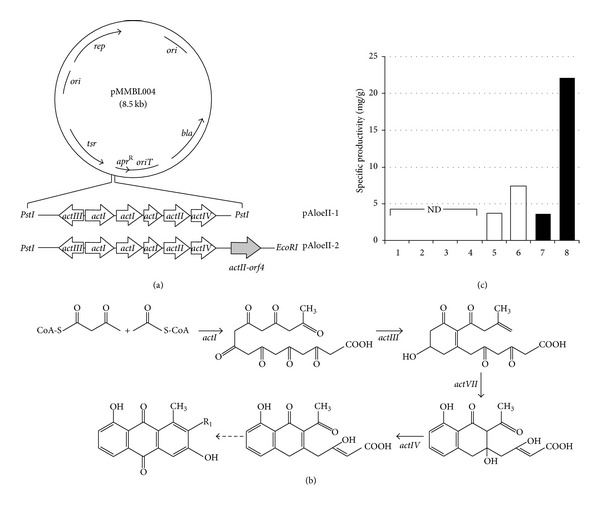
(a) Plasmid map of the pMMBL004, pAloeII-1 (pMMBL004 containing *Pst*I fragment), and pAloeII-2 (pAloeII-1 containing *actII-orf4*); *rep*: replicon; *ori*: origin of replication; *bla*: *β*-lactamase^R^; *tsr*: apramycin^R^; *apr*: apramycin^R^; *oriT*: origin of transfer. (b) Schematic representation of the aloesaponarin II biosynthetic pathway. Solid and dotted arrows represent presumed enzymatic and spontaneous steps, respectively. 3,8-DMAC: 3,8-dihydroxy-1-methyl-anthraquinone-2-carboxylic acid. R1 groups of aloesaponarin II and 3,8-DMAC are hydrogen and carboxylic acid, respectively. (c) Productions of aloesaponarin II and 3,8-DMAC in recombinant *S. coelicolor* strains. 1, *S. coelicolor* ΔACT/pMMBL004; 2, *S. coelicolor* ΔACTΔ*wblA*ΔSCO1712ΔSCO5426/pMMBL004; 3, *S. coelicolor* ΔACT/pAloeII-1; 4, *S. coelicolor* ΔACTΔ*wblA*ΔSCO1712ΔSCO5426/pAloeII-1; 5, *S. coelicolor* ΔACT/pAloeII-2; 6, *S. coelicolor* ΔACTΔ*wblA*ΔSCO1712ΔSCO5426/pAloeII-2; 7, *S. coelicolor* ΔACT/pAloeII-2; 8, *S. coelicolor* ΔACTΔ*wblA*ΔSCO1712ΔSCO5426/pAloeII-2; 1~6, liquid culture; 7~8, solid culture; ND, not detected [29].

 A polyketide nonproducing *S. coelicolor* mutant strain was generated by deleting the entire actinorhodin cluster from the chromosome of a previously generated *S. coelicolor* Δ*wblA*ΔSCO1712ΔSCO5426 triple knock-out mutant strain, which was shown to stimulate actinorhodin biosynthesis through deletion of 2 antibiotic down-regulators as well as a polyketide precursor flux downregulator [47]. Using this engineered *S. coelicolor* mutant strain as a surrogate host or a cell factory from a synthetic biology perspective, a model minimal polyketide pathway for aloesaponarin II [48] was cloned and functionally expressed in a high-copy expression plasmid, followed by quantitative polyketide analysis. As expected, aloesaponarin II production was observed at the highest level in the actinorhodin cluster-deleted and downregulators-deleted mutant strain,* S. coelicolor* ΔACTΔ*wblA*ΔSCO1712ΔSCO5426 (Figure 4). These results imply that this engineered actinorhodin-free and regulation-optimized *S. coelicolor* mutant strain can be used as a general surrogate for efficiently expressing foreign polyketide pathways. In conclusion, biotechnological applications of the independently functioning regulatory pathway identified through microarray-driven reverse engineering strategy may be beneficial for *Streptomyces* strain improvement for polyketide overproduction as well as for efficient host cell factory construction for synthetic biology.

## Figures and Tables

**Table 1:** *wblA* orthologs identified from various *Streptomyces* species.